# Small Water Enterprise in Rural Rwanda: Business Development and Year-One Performance Evaluation of Nine Water Kiosks at Health Care Facilities

**DOI:** 10.3390/ijerph14121584

**Published:** 2017-12-16

**Authors:** Alexandra Huttinger, Laura Brunson, Christine L. Moe, Kristin Roha, Providence Ngirimpuhwe, Leodomir Mfura, Felix Kayigamba, Philbert Ciza, Robert Dreibelbis

**Affiliations:** 1 The Center for Global Safe Water, Sanitation and Hygiene, Rollins School of Public Health, Emory University, Atlanta, GA 30322, USA; lbrunson7@gmail.com (L.B.); clmoe@emory.edu (C.L.M.); kmroha@gmail.com (K.R.); 2 The Access Project Rwanda, Kigali, Nyarugenge District, Rwanda; ramond2020@gmail.com (P.N.); leodomir2003@gmail.com (L.M.); fkaigamba@gmail.com (F.K.); 3 The Republic of Rwanda Ministry of Health, Environmental Health Desk, Kigali, Kicukiro District, Rwanda; cizaphilbert@gmail.com; 4 School of Civil Engineering and Environmental Science, The University of Oklahoma, 4, Norman, OK 73019, USA; Robert.Dreibelbis@lshtm.ac.uk

**Keywords:** cost model, demand estimation, water treatment, sub-Saharan Africa

## Abstract

Small water enterprises (SWEs) have lower capital expenditures than centralized systems, offering decentralized solutions for rural markets. This study evaluated SWEs in rural Rwanda, where nine health care facilities (HCF) owned and operated water kiosks supplying water from onsite water treatment systems (WTS). SWEs were monitored for 12 months. Spearman’s Rank Correlation Coefficient (r_s_) was used to evaluate correlations between demand for kiosk water and community characteristics, and between kiosk profit and factors influencing the cost model. On average, SWEs distributed 15,300 L/month. One SWE ran at a loss, four had profit margins of ≤10% and four had profit margins of 45–75%. Factors influencing SWE performance were intermittent water supply (87% of SWE closures were due to water shortage), consumer demand (demand was high where populations already used improved water sources (r_s_ = 0.81, *p* = 0.02)), price sensitivity (demand was lower where SWEs had high prices (r_s_ = −0.65, *p* = 0.08)), and production cost (water utility tariffs negatively impacted SWE profits (r_s_ = −0.52, *p* < 0.01)). Sustainability was more favorable in circumstances where recovery of capital expenditures was not expected, and the demand for treated water was sufficient to fund operational expenditures. Future research is needed to assess the extent to which kiosk revenue can support ongoing operational costs of WTS and kiosks both at HCF and in other contexts.

## 1. Introduction

Small water enterprises (SWEs) are decentralized water service providers that extend water provision beyond the reach of water utilities [1,2]. SWEs have become ubiquitous in low- and middle-income countries, particularly among urban populations that are un- or under-served by utilities [3,4,5,6,7,8,9,10]. Although there is a large diversity in SWEs, distributing vendors and direct vendors are the two broad types that dominate water distribution in low- and middle-income countries [2,3,4,5,8,9,10,11,12,13,14,15,16,17]. Distributing vendors take water to the consumer through tanker trucks, carts, or delivery systems, while direct vendors have a central location that consumers visit to obtain water [17]. Water kiosks, one variant of the direct vendor model, are stationary water points where operators oversee container filling and collect payment. Water kiosks may be extensions of a public utility or have an independent water source, such as a borehole, and can be owned and operated by private enterprises, community-based groups, NGOs, or hybrids of those entities [1].

For SWEs, demand for safe water in the target population, competition with other vendors, and generation of revenue to fund operation and maintenance are critical factors for financial sustainability [5,7,18,19]. SWEs must operate consistently in order to reliably provide water to meet consumer demand because a lack of continuity in water supply can drive consumers to choose alternative sources [20,21]. As SWEs have become increasingly visible in urban markets in low-income countries, both private start-ups and donor-funded programs have attempted to leverage this success to extend safe water kiosk programs to rural areas, where significant unmet need for safe drinking water persists [22,23,24,25,26,27,28]. However, rural locations have proven difficult for SWEs, due to low population densities, small disposable incomes in the potential user population, intermittent utility supply, and low capacity to maintain infrastructure and supply chains [7,21,29,30].

Information on the viability of SWEs in rural areas is predominantly in grey literature, such as program reports, and there are limited critical analyses of SWE feasibility and functionality in rural contexts [2,10,17]. This study examined rural SWE operations in Rwanda, including market analysis, demand estimation, production costs, consumer demand, kiosk revenue, and the feasibility of recovering operational expenditures for the water treatment systems (WTS). A secondary objective of this study was to assess the viability of placing kiosks at heath care facilities (HCF) with HCF staff managing kiosk operation.

## 2. Materials and Methods

Between March and December of 2012, water treatment systems (WTS) using membrane ultrafiltration and chlorination were donated and installed in ten rural health care facilities (HCF) located in two districts of Rwanda. The ten HCF selected for participation in the program were nominated by the Rwanda Ministry of Health, and met inclusion criteria of having multiple on-site water sources (piped water supply and rainwater catchment); water quality that did not meet WHO drinking water guidelines; a reliable power supply; and were willing to dedicate staff effort to WTS operation, maintenance and kiosk operation [31]. The value of each WTS was approximately 15,000 USD, and the peak output capacity was estimated by the manufacturer to be 50,000 L per day [31]. HCF staff were trained in daily and weekly operations and maintenance of the WTS. On-going support, including WTS servicing and repair, was provided by the implementing organization from installation in 2012 until handover in 2015. This support included routine visits by local contractors and periodic visits by staff from the implementing organization. An in-depth, prospective evaluation of WTS performance at the HCF is presented in Huttinger et al. [31]. Participating HCF were provided the option of establishing a kiosk to sell treated water to households in surrounding communities. All ten HCF with WTS decided to open kiosks, however, nine of ten are included in this article because the last kiosk opened after the study period had concluded.

This article describes the phases of implementation of nine HCF-based, community-serving kiosks and evaluates the kiosk operation and financial performance. Study activities occurred between March 2011 and August 2014 and consisted of three distinct phases: market analysis and demand estimation in 2011–2012, treated water production cost estimation and intervention development in 2012–2013, and monitoring of kiosk operation and evaluation of financial performance in 2013–2014. Study activities were iterative, and each informed the delivery of the next. The relationships between kiosk operation, performance and community characteristics were assessed. The advantages of placing kiosks at HCF, and the additional burden to HCF, are discussed.

### 2.1. Market Analysis and Demand Estimation

An analysis of the market for water was conducted in communities surrounding the HCF to assess the extent to which HCF-based kiosks could be a competitive alternative to existing water sources. The analysis consisted of a household survey and a census of water sources conducted in the villages surrounding the ten HCF where WTS were implemented. The household survey, administered by trained enumerators, addressed household water collection (where and how people collected water) and water treatment and storage practices in a random sample of 25–35 households living within 1 km of each of the participating health facilities, for a total sample of 312 households. Data were entered using Access (Microsoft Corporation, Redmond, WA, USA) and analyzed using SAS (SAS Institute, Cary, NC, USA). Second, a census of water sources identified all sources within 2 km of each HCF and documented source type, functionality, and the cost of water, where applicable. Data were entered and analyzed using Excel (Microsoft Corporation, Redmond, WA, USA). Data collection was complemented with a literature and policy scoping exercise to reference national guidelines for improved water source access and, where possible, district- and sector-specific information about population size, per capita water consumption, access to improved water sources (distance to and type of source), and household water treatment practices. Searches were conducted using Web of Science and Google Scholar in an iterative manner to identify articles, reports and policy documents related to household water collection and treatment practices in Rwanda. Market analysis and demand estimation informed the cost model and implementation of the kiosks, and served as a starting point to evaluate the extent to which the target population used the HCF kiosks as water source. Correlations between consumer demand and community characteristics were examined using non-parametric Spearman’s Rank Correlation Coefficient (r_s_). Data were analyzed using SAS (SAS Institute, Cary, NC, USA).

### 2.2. Production Cost Estimation and Implementation

Estimated production cost was the cost of WTS operation to produce treated water. Capital expenditures and repair and replacement costs were provided by the donor for a three-year period following WTS donation, therefore, capital expenditures for repairs were not observed nor were estimates included in the cost model. 

Participatory methods were used to establish an equitable price for purified water at each kiosk; program staff and a representative from the Ministry of Health presented cost model projections and local market analyses and facilitated discussions among community leaders and health center advisory committees. Participating HCF were given the choice to accept or reject the kiosk, and were free to set their own prices and operating hours and assign operating personnel. Implementation of the kiosks followed a uniform design and launch. Branded advertising and promotion were developed with input from HCF staff and community health workers, and were approved by HCF leadership and the Ministry of Health. Trainings were provided by the Rwandan implementing partner in coordination with the Ministry of Health to community health workers to promote safe water source selection, transport, and storage in the communities surrounding the HCF. Trainings led by the implementing partner were delivered at HCF to prepare staff to perform financial and operations management of the kiosks. The authors contributed to the development of training materials.

Prior to kiosk opening, training was provided to Community Health Worker (CHW) Supervisors and Environmental Health Officers (EHOs) at the HCF addressing interpersonal communication (IPC) for behavior change to promote safe water source selection, transport and storage in order to build community awareness of the importance of safe drinking water. Trainings were delivered by the implementing organization, in coordination with the Ministry of Health. Twenty-five CHW supervisors and EHOs trained 280 CHWs, and distributed IPC support materials (flipcharts, booklets). Trainings were conducted, on average, one month before the kiosks were opened and CHWs incorporated the information into routine community-based behavior change education to approximately 5000 people in the 30 villages that were within a radius of 1 km from HCF. Kiosks were opened with official ceremonies organized by the HCF with participation from community leaders, and the first 100 customers to purchase water from each kiosk were given a branded 10 L water container. Follow-up on community education was conducted via monthly meetings at the HCF that continued for up to 6 months after the kiosks opened to encourage CHWs to promote IPC for behavior change for safe water practices. At the HCF, during health awareness sessions led by EHOs for patients and visitors, safe water practices were addressed at least once per week for up to 6 months after the kiosks opened. These actions were coordinated by the implementing organization with support from the Ministry of Health.

### 2.3. Monitoring of Kiosk Operation, Consumer Demand, and Evaluation of Financial Performance

The implementing organization conducted monthly visits to discuss kiosk performance with the HCF staff, and promote good practices for financial management and efficient water use for 6–12 months following kiosk opening; duration of follow-up depended on when each kiosk was launched within the span of the study. Kiosk operators were trained to record water meter readings each day and record volumes of water treated and distributed to the HCF and kiosk. Revenue generated from the kiosk, reasons for kiosk closures, and water meter readings were recorded daily onto a standardized form. Daily records of kiosk operations between August 2013 and August 2014 were shared with study researchers on a monthly basis, and data were double entered into an electronic database (Open Data Kit, opendatakit.org) and analyzed using SAS (SAS Institute, Cary, NC, USA) and Excel (Microsoft Corp., Redmond, WA, USA). Consumer demand was measured as the volume of water distributed from the HCF kiosks. Observed revenue was cash received at the kiosk, recorded in kiosk operator logs, and verified by HCF accountants. Production cost was calculated by (i) measuring water consumption, including rainwater, via water meter readings, (ii) tracking the tariff charged by the water utility, and (iii) by estimating the costs of power (cost of electricity to run water pumps based on kwh consumption and estimated run time) and chlorine (cost of chlorine used for post-filtration disinfection of treated water) for volume of water treated. Profit was calculated by subtracting the production cost for the volume of water distributed to the kiosk from the observed revenue; profit margin (%) was calculated by dividing profit by observed revenue. Projected revenue was calculated by multiplying the volume of water distributed from the kiosk by the treated water sale price. Loss was calculated as the difference between projected revenue and observed revenue, with an adjustment for water loss due to cleaning and spills which was estimated to be 8% or 1.6 L lost for every 20 L dispensed. Correlations between demand for treated water from kiosks and community characteristics, and between kiosk profit and factors influencing production cost were examined using non-parametric Spearman’s Rank Correlation Coefficient (r_s_).

### 2.4. Ethical Approval

The study was reviewed and approved by the Institutional Review Board at Emory University (No. IRB00053040, as amended) and the Rwanda National Ethics Committee (No. 646/RNEC/2014).

## 3. Results

### 3.1. Market Analysis and Demand Estimation

#### 3.1.1. Market Analysis

The household survey revealed that 81% of households living within 1 km of the HCF used a public tap as their primary water source (range: 62–100% per site). Households spent, on average, 35 Rwandan Francs (equivalent to 0.05 USD [32]), per day, on water (SD: 28 Rwandan Francs). Distance to water source was the most commonly reported factor (58%) influencing water source selection, compared to perceived cleanliness/safety of the water (32%) and price (10%). The average travel time to the primary water source was 14 min (SD: 15 min). The census of water sources found that the number of national utility water sources within 2 km of HCF ranged from 2 to 23; the number of national utility water sources within 500 m of HCF ranged from 0 to 5. Average costs at national utility water sources ranged from 10 to 20 RWF (0.02–0.03 USD) per 20 L container (Table 1).

#### 3.1.2. Demand Estimation

The literature and policy scoping exercise found policy documents addressing improved water source access and per capita consumption, nationally representative studies with population demographics by district, water source type and distance to primary water source, and reports from district-level programs that addressed household water treatment [33,34,35,36,37,38,39,40]. The Government of Rwanda defined improved water access as an improved water source within 500 m, while the World Health Organization global guideline was within 1 km [23,38]. Per capita water consumption was reported by the Government of Rwanda to be 8 L per day in rural areas [37]. District-specific reports found that 50% and 79% of households reported using a standpipe as their primary water source, and 48% and 38% of households were within 500 m of their primary water source, respectively, in the districts located in Northern Province and Eastern Province, and up to 25% of households in both districts reported household drinking water treatment using boiling, filtration, or chlorination [36,38,39,40]. Population data from the national census was disaggregated to the smallest level available [41].

Demand estimates were derived from population size, distance from HCF, primary water source type, distance to primary water source, household water treatment, and per capita water consumption. The national estimate for rural per capita water consumption of 8 L per person per day was used [37]. The resulting demand is presented in Table 1. Demand estimation assumptions, calculation and site-specific information are presented in the supplementary material.

### 3.2. Cost Model and Implementation

The estimated production cost for treated water was 14 RWF (0.02 USD [32]) for 20 L; this cost decreased when rainwater was used to supplement purchased national utility water, with 50% rainwater use resulting in an estimated production cost of 9 RWF (0.01 USD) for 20 L, equivalent to 1.05 and 0.68 USD per m^3^, respectively. Kiosk water prices proposed by the HCF and community representatives ranged from 20 to 50 RWF (0.03–0.08 USD) per 20 L container; equivalent to 1.50–3.50 USD per m^3^. HCF directors set the final treated water price at each HCF, and unanimously decided that kiosk operation would be included in the duties of the HCF staff without additional compensation, thus, the cost model was adjusted to exclude labor cost. Nine of the ten planned kiosks were launched during the observation period. The opening of the kiosk at the tenth site was delayed due to weather damage that was resolved after the study had concluded.

### 3.3. Kiosk Operation and Financial Performance

#### 3.3.1. Kiosk Operation

Observation time at the nine sites ranged from 2–12 months, depending on when each kiosk opened. Kiosk operation, defined as the kiosk being open and selling water, ranged from continuous (100%) to 30% of the observation period (Table 2). A total of 50 cumulative months of observation were available to the study team, and observations conducted over a period of 12 months were used for the analyses (Table 2). Two thirds of HCF experienced one or more water shortages that resulted in kiosk closure, where closure was defined as no kiosk operation during a one-day period. Overall, HCF were able to provide manpower and oversight for WTS and kiosk operations, and financial management. The HCF paid utility bills and maintained on-site infrastructure to allow WTS use and distribution of treated water to the kiosk, when water was available.

#### 3.3.2. Consumer Demand and Financial Performance 

**Figure 1:**
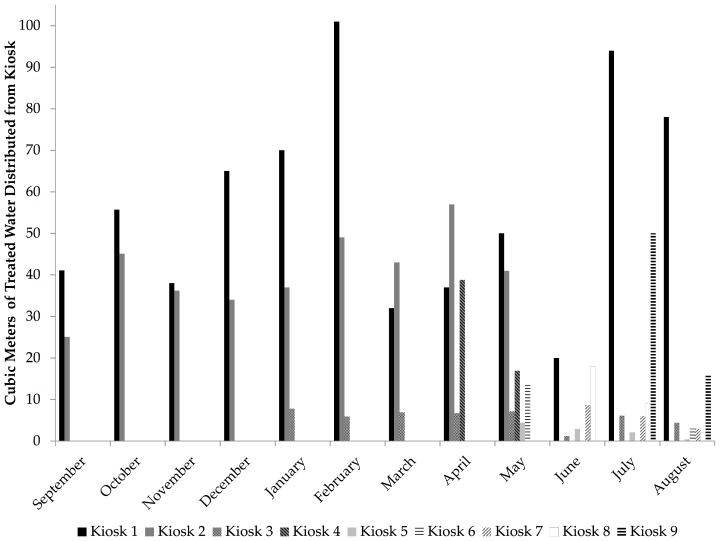
Volume of Water Distributed from Kiosk per Month at Nine Water Kiosks at Health Care Facilities in Rural Rwanda, 2013–2014.

Consumer demand for treated water from the kiosks ranged from 2–57 m^3^ per kiosk per month (Figure 1). This averaged to 700 L per kiosk per day (Supplementary Figure S1). Consumer demand was five times lower than the demand estimated by the model (Table 1). The number of public piped water sources within 2 km of the HCF kiosk had the strongest relationship with consumer demand (r_s_ = 0.81, *p* = 0.02). Whereas, there was no relationship between consumer demand and the number of public piped water sources within 500 m of the HCF kiosk (r_s_ = 0.27, *p* = 0.48) (Table 1). There was negative correlation (r_s_ = −0.65, *p* = 0.08) between consumer demand and higher price at the HCF kiosks compared to other sources, but this was not significant (Table 1). The kiosks that sold treated water at more than twice the price of other public piped water sources had the lowest consumer demand (Kiosks 5, 6). There was no correlation between consumer demand and the size of the population within one kilometer of the kiosk (r_s_ = 0.41, *p* = 0.24) (Table 1).

Observed revenue from sales of treated water at the kiosks was on average 1.51 USD/m^3^ (range 0.83–2.56 USD) per month (Table 3). Production cost was on average 1 USD/m^3^ (range 0.53–1.27 USD) (Table 3), excluding labor and capital expenses. The cost of purchasing piped water from the utility accounted for 90% of the production cost on average (range: 76–96%). At 6 out of 9 HCF, it was possible for rainwater to be integrated into the WTS water supply, and the amount of rainwater used among these sites ranged from 7% to 41%, depending on rainfall, and capture and storage capacity (Table 3).

**Figure 2:**
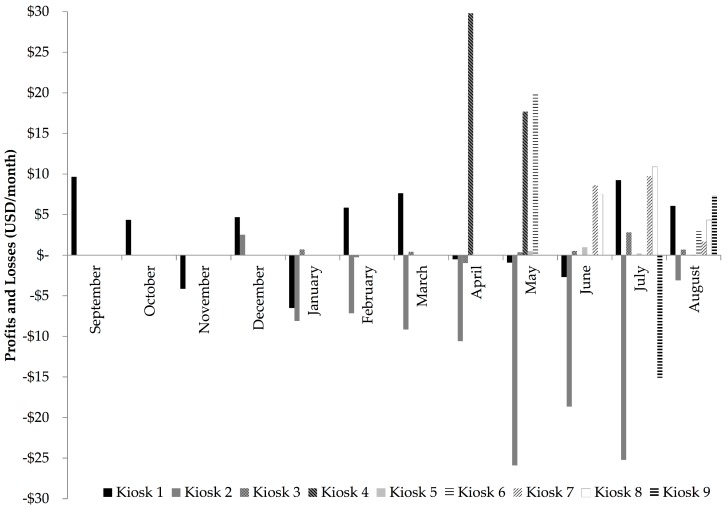
Monthly Profits and Losses of Nine Water Kiosks at Health Care Facilities in Rural Rwanda, 2013–2014.

All kiosks experienced increases and decreases in observed revenue over the course of the study (Figure 2). The profit or loss per month per kiosk ranged from −26 to 30 USD (Figure 1). The overall average was 4 USD profit per kiosk per month (Table 4). Substantial losses were observed at Kiosk 2 throughout the observation period (Figure 1). When this outlier kiosk was removed from analysis, the average profit rose to 6 USD per kiosk per month (Table 4). Averaging across the observation period, the profit margin was 25% increased return on operations costs (Table 3). The median profit margin was 10%. Overall, four kiosks had 45–75% increase in profit margin, four kiosks had nominal increases of ≤10% in profit margin, and one kiosk (Kiosk 2) experienced financial losses for the duration of the observation period, and had the greatest water loss (Table 4).

#### 3.3.3. Measures of Correlation

Profit had an inverse relationship with water loss (r_s_ = −0.66, *p* < 0.01), production cost (r_s_ = −0.52, *p* < 0.01), and volume of water distributed from the kiosk (r_s_ = −0.51, *p* < 0.01), (Table 3). There was not a significant association between profit and use of rainwater (r_s_ = 0.21, *p* = 0.25), (Table 3).

## 4. Discussion

This study provides contextual information and detailed analysis of the operational and financial performance of this SWE model in the first year of operation. In general, HCF were able to provide the necessary staff to operate the kiosk, and repair needs resulted in minimal loss of operational time due to WTS problems, however, there were substantial water shortages that affected the overall operational performance of the SWEs. Despite having met the study inclusion criteria of having multiple on-site water sources, the majority of HCF (six of nine) experienced intermittent kiosk operation during the observation period. Irregular kiosk operation due to water shortage may have contributed to lower than predicted demand. In Kenya, gaps in water provision from SWEs led to customer dissatisfaction [15,16]. Considering that in rural contexts, populations typically use a variety of water supplies as opposed to dependence on a single source, unreliable water provision from the kiosks may have driven people to use other sources, including unimproved water sources [42]. Intermittent water supply negatively impacted the performance of these kiosks, and is a factor that should be considered in future SWE programs, particularly where success is dependent on economies of scale and large quantities of water must be sold for the SWEs to continue to be viable. Intermittent water supply and the length of the observation period at each kiosk limited the extent to which this study could evaluate seasonal variation on demand from water kiosks. At Kiosk 1, decreased demand was observed during the rainy season (March–June), however, similar trends were not observed at Kiosk 2 or 3, and Kiosks 4–9 were not observed for long enough to identify seasonal variation.

Averaging across all kiosks, twenty percent of the expected demand was met by the kiosks, which is comparable to the results of trials independently conducted to evaluate consumer demand for rural SWEs that have found 10–38% of target populations have purchased water from kiosks [2,10,15]. The method used in this study to measure consumer demand did not differentiate the volumes of water purchased by kiosk users—we do not know if there were a few people who purchased large volumes of water, or if many people purchased smaller quantities. The model for estimated demand assumed 8 L/person/day following information provided by the Government of Rwanda, which was comparable to other estimates [21,37]. Field research has indicated that per capita use of water from SWEs is much lower, typically 2–3 L [15,28]. If the demand estimate calculations made had used an assumption of 2 L/person/day, rather than 8 L/person/day, then the volumes of water that were sold would have reached on average of 80% of target population, or approximately 23% of the population living within 1 km of the HCF. (Supplementary Table S1). It is important that future models predicting SWE profitability and sustainability consider that per capita demand for treated water will be low, particularly where populations are already served by other improved water sources or where lower-cost alternatives, including unimproved sources, are available.

The greatest consumer demand was observed at kiosks in communities where there were other improved piped water sources, and where the kiosk price was competitive with those sources. The least demand was observed at kiosks in communities where there were fewer improved piped water sources, and the kiosk price was double the price of the other sources. This indicates that consumers were sensitive to cost, as has been documented in Ghana and Kenya [15,28,42,43]. This also indicates that demand creation was the most successful in locations where the population was accustomed to collecting water from improved sources and paying for water. This has important implications for the target markets for future kiosks, particularly in rural areas. In contexts where government and/or international development programs have subsidized water costs, and in contexts where community water committees periodically collect user fees as opposed to paying for water at time of collection, or where primary water sources are free, SWEs present a shift in the way households spend money on water.

Distance to water source was the most influential factor in water source selection in the target population, and other studies of water source selection have described similar outcomes [16,27,42]. Efforts to increase consumer demand by marketing to a larger (and more geographically disperse) population are unlikely to be successful if kiosk users are expected travel longer distances than they would to reach another improved water source. SWEs that have opened satellite retail vendors, and have expanded operation to include water delivery, have been able to overcome this hurdle [14,44,45].

A key requirement shared by independent, viable SWEs is that they are financially sustainable and recover their costs [18]. Safe Water Network has found that financial sustainability of rural SWEs in India, Ghana, and Kenya depended upon competitive pricing, as well as a greater volume of water sold, since the unit production costs declined as sales volumes decreased, due to economies of scale [13,22,44,45]. However, among the HCF kiosks in Rwanda, there was an inverse relationship between sales volume and production cost that was driven by the cost of water. Government facilities (including hospitals, health centers, and schools) were subject to a 61% tariff increase for consuming more than 50 m^3^ per month, and a 14% tariff increase for consuming more than 100 m^3^ [46]. This resulted in greater costs for water purchased by the HCF, including for kiosk use. Non-punitive tariffs would have improved kiosk revenue, but policy change was beyond the scope of the project [46,47]. Decoupling HCF and kiosk water supplies would have reduced the pressures posed by the water tariff, but the WTS set-up used the HCF water storage tanks for both the HCF and the kiosks, which would have made this change technically undesirable, because of the observed water shortage problems.

One site, Kiosk 2, was an outlier in that it had persistent financial losses. There were two factors at this site that differentiated it from the others: the kiosk operators provided water on credit to customers, and there were leaks in the kiosk piped water infrastructure that took months to identify and repair. Offering short-term credit to customers has been presented as a benefit of SWEs in serving poor populations, however, this strategy may have contributed to the substantial losses at Kiosk 2 [1,11]. At all sites, the cost of labor was excluded from cost estimates because the HCF absorbed staff costs. Profits generated by sales could not have covered part-time unskilled labor for kiosk operation. Furthermore, if consumer demand for safe water from the kiosks increased, the HCF may need to hire kiosk operators to relieve the increased labor burden on HCF staff. Additional staff costs would substantially, if not completely, reduce the profitability of the kiosks. Due to the water tariff structure, an increase in consumer demand would result in further reduction of profitability, due to increased production cost. The donor-assisted implementation and the short observation period resulted in an inability for this study to capture capital maintenance expenditures and factor them into the cost model.

## 5. Conclusions

The SWE model documented in this study was enabled by the donation of a WTS, and leveraged existing HCF infrastructure for rainwater collection and water storage. These capital expenditures are typically the most expensive component of a SWE. WTS designed for SWE application are substantially less costly than large-scale WTS, however, the likelihood of WTS cost recovery in most rural contexts in sub-Saharan Africa is small, as evidenced from case studies in Kenya, Ghana, and this study in Rwanda [15,28,42,43]. SWE sustainability is more favorable in circumstances where recovery of capital expenditures is not expected, and the demand for treated water is sufficient to fund operational expenditures, including labor. This SWE model, where the HCF staff provided kiosk labor, allowed for the kiosk to generate small profit, despite low consumer demand. There are two factors that provide limited evidence that SWEs can operate in certain contexts in rural sub-Saharan Africa. First, four of nine HCF kiosks returned 45–75% profit per month during year one; second, other case studies of rural safe water kiosk SWEs have documented increases in consumer demand beyond year one. Continued operation for many years may be impeded by needs for capital for maintenance and repairs. Because operational expenses associated with labor present such a burden for small-scale operations, SWEs may not be able to generate sufficient revenue to fund maintenance and repairs. In this study, kiosk revenue was insufficient to fund labor and WTS repair and replacement costs. High price sensitivity and low demand for treated water in rural contexts exacerbate the risks of kiosks generating insufficient funds. Even in scenarios where recovery of capital expenditures is not expected, careful consideration must be given to the extent to which kiosk revenue can support ongoing operational expenses and help ensure sustainability of these donated WTS in healthcare facilities—a key objective of the donor.

## Figures and Tables

**Table 1:** Community Characteristics in the Vicinities of Nine Health Care Facilities in Rural Rwanda 2011–2012, Demand for Treated Water from Kiosks Located at the Health Care Facilities, 2013–2014, and Measures of Correlation.

Characteristic	Mean	Range	r_s_^†^	*p* Value
Observed demand for water per kiosk per month (m^3^)	21	(2–57)	(*Ref.*)	
Size of population within 1 km of kiosk	1967	(609–4045)	0.41	0.24
National utility water sources within 2 km of kiosk	9	(2–23)	0.81	0.02
National utility water sources within 500 m of kiosk	3	(0–5)	0.27	0.48
Increase in price of water from national utility sources to kiosk (%) ^1^	83	(−38–214)	−0.65	0.08
Estimated demand for water per kiosk per month (m^3^)	116	(57–205)	*-*	
Estimated demand met per month (% observed/estimated)	20	(3–49)	*-*	

^†^ Spearman’s Rank Correlation Coefficient (r_s_) was used to measure the extent and significance (*p* value) of correlation between community characteristics (rows 2–5) and observed demand for water per kiosk per month (row 1), the reference value (*Ref.*). ^1^ The cost of water from national utility sources ranged from 10 to 20 RWF (0.02–0.03 USD) per 20 L container.

**Table 2:** Water Kiosk Operation and Closure, and Reasons for Closures at Nine Health Care Facilities in Rural Rwanda, 2013–2014.

Kiosk	1	2	3	4	5	6	7	8	9	Mean
Months of observation	12	9	8	5	4	4	3	3	2	5.5
Percent of observation period when kiosk was closed	10	0	10	70	60	70	0	50	0	30
Percent of closure time due to water shortage	80	0	90	100	83	86	0	80	0	86
Percent of closure time due to offline water treatment system	10	0	0	0	17	0	0	0	0	7
Percent of closure time due to lack of manpower	10	0	10	0	0	14	0	20	0	7

**Table 3:** Month Averages per Kiosk for Revenue, Profit Margin, Volume of Water Distributed, Production Cost, Water Loss and Measures of Correlation, for Nine Water Kiosks at Health Care Facilities in Rural Rwanda, 2013–2014.

Kiosk	1	2	3	4	5	6	7	8	9	Mean	r_s_^†^	*p* Value
Profit margin per m^3^ sold (%)	3	−34	10	54	7	74	56	47	4	25	(*Ref.*)	
Volume distributed (m^3^)	57	41	6	28	3	8	6	10	33	21	−0.51	<0.01
Rainwater used (%)	7	22	0	41	0	30	14	22	0	23	0.21	0.25
Production cost (USD/m^3^)	1.03	1.11	1.14	0.78	1.27	0.53	0.79	1.24	1.09	1.00	−0.52	<0.01
Water loss per m^3^ sold (%)	19	53	5	15	30	10	8	6	12	18	−0.66	<0.01

^†^ Spearman’s Rank Correlation Coefficient (r_s_) was used to measure the extent and significance (*p* value) of correlations between kiosk characteristics (rows 2–5) and profit from treated water sales at the kiosks (row 1), the reference value (*Ref.*).

**Table 4:** Total Production Volumes, Kiosk Profits, and Costs of Water Treatment for Nine Health Care Facilities and Water Kiosks in Rural Rwanda, 2013–2014.

Kiosk	1	2	3	4	5	6	7	8	9
Total m^3^ of treated water distributed to kiosk and HCF	1383	1137	541	93	124	168	280	178	132
Total production cost (USD) of treated water distributed to kiosk and HCF	1430	1224	644	80	167	135	271	220	155
Total m^3^ treated water distributed to kiosk (% total volume)	739 (53)	367 (32)	46 (9)	56 (60)	1 (1)	16 (10)	18 (6)	29 (16)	66 (50)
Total production cost of treated water distributed to kiosk (USD) *	766	416	54	47	12	7	13	35	81
Total kiosk revenue (USD)	802	310	59	95	14	30	33	58	73
Total kiosk profit (USD)	36	−106	5	48	2	23	20	23	−8
Average kiosk profit * (USD/month) (range)	3 (−7–10)	−12 (−26–3)	1 (−1–3)	24 (0–68)	0 (0–1)	12 (0–20)	7 (2–10)	8 (4–11)	−4 (−15–7)

* Average kiosk profit across the nine sites was 6 USD/month. Average kiosk profit for all sites excluding Kiosk 2 was 4 USD/month.

## Supplementary Materials

The following are available online at www.mdpi.com/1660-4601/14/12/1584/s1, Figure S1: Estimated Demand for Treated Water from HCF kiosk and Influencing Factors, Table S1: Estimated Demand Calculation and Assumptions.
