# Supplementary material for: Small Water Enterprise in Rural Rwanda: Business Development and Year-One Performance Evaluation of Nine Water Kiosks at Health Care Facilities
Source: Int J Environ Res Public Health. 2017 Dec 16;14(12):1584. doi: 10.3390/ijerph14121584 (PMC5751002; doi:10.3390/ijerph14121584)
Supplement: Supplementary file 1 [file ijerph-14-01584-s001.pdf]

Figure S1. Estimated Demand Calculation

$$\mathrm{Pop_{kiosk}} = \mathrm{Prop_{pay\ water}} * \left(\left(\begin{array}{c} \mathrm{Pop_{500m-1km}} \\ - \\ \mathrm{Pop_{500m-1km}} * \mathrm{Prop_{water\ 500m}} \\ - \\ \mathrm{Pop_{500m-1km}} * \mathrm{Prop_{treat\ water}} \end{array} \right) + \left(\begin{array}{c} \mathrm{Pop_{>500m}} \\ - \\ \mathrm{Pop_{>500m}} * 0.5 * \mathrm{Prop_{treat\ water}} \end{array} \right) \right)$$

$\mathrm{Pop_{kiosk}}$ *Population estimated to be kiosk users*

$\mathrm{Prop_{pay\ water}}$ *Household primary water source is a standpipe (%)* [2]

$\mathrm{Pop_{500m\text{-}1km}}$ *Population between 500m and 1km from HCF* [5]

$\mathrm{Prop_{water\ 500m}}$ *Population within 500m of primary water source* [2]

$\mathrm{Prop_{treat\ water}}$ *Household water treatment (%)* [3,4]

$\mathrm{Pop_{>500m}}$ *Population 500m or less from HCF* [5]

Estimated Demand Assumptions

Households located further than 1km from the HCF were not included in demand estimation for two reasons. First, household surveys showed that distance to primary water source was a critical factor in water source selection. Second, average reported travel time to the primary water source was less than 15 minutes - generally equivalent to the time to walk 1km. It was assumed that only households that already used a standpipe as their primary water source – and therefore paid for water as opposed to collecting it for free – would collect water from the HCF kiosk. Among households located between 1km and 500m from a HCF, it was assumed that households located 500m or less from their primary water source would not have an incentive to travel further to collect water from the HCF kiosk and were thus excluded. It was also assumed that households who already reported treating their water would not be willing to collect water from the HCF since the kiosk water may not have been perceived as being of better quality and were thus excluded. Among households located 500m or less from a HCF, it was assumed that the households who did not report treating their water would be motivated to collect water from the HCF kiosk because it would be a similar or shorter distance away than their primary water source and of better water quality. Finally, it was assumed that among households who reported treating their water that about 50 % would find it more convenient to collect water from the HCF kiosk than to spend time and money to treat water. The size of the population within 1km of the health center was derived from cell-level census data and ranged from 609 to 4054 per site (mean: 1967).

Table S1. Estimated Demand for treated water from HCF kiosk and influencing factors

National Estimates	National									
Rural per capital consumption of water (liters/person/day)[1]	8									
**Regional Estimates**	**Distrtict 1 (Northern Province)**					**District 2 (Eastern Province)**				
Population within 500m of primary water source (%) [2]	48					38				
Household primary water source is a standpipe (%) [2]	50					79				
Hosuehold water treatment (%) [3,4]	25					25				
**Site-specific Estimates for HCF**	**1**	**2**	**7**	**8**	**9**	**3**	**4**	**5**	**6**	**Average**
Population within 1 km of HCF [5]	3696	2495	2219	4054	1761	977	609	949	945	1967
Population estimated to be kiosk users	778	525	467	853	370	383	238	372	370	484
Popualtion with 1km of HCF estimated to be kiosk users (%)	21	21	21	21	21	39	39	39	39	29
Estimated monthly demand assuming 8 liters/person/day (m^3)	187	126	112	205	89	92	57	89	89	116
Actual demand month average (m^3)	57	41	6	10	33	6	28	3	8	21
Estimated demand that was met at 8 liters/person/day (%)	30	32	5	5	37	6	49	3	9	20
Estimated demand that was met 2 liters/person/day (%)	122	129	22	19	148	25	194	11	36	78

[1] Republic of Rwanda Ministry of Infrastructure, National Policy and Strategy for Water Supply and Sanitation Services. Kigali, Rwanda, 2010.
[2] National Institute of Statistics of Rwanda, *Integrated Household Living Conditions Survey (EICV3 2010/11)*. Kigali, Rwanda, 2011.
[3] Population Services International, Scaling Up Point-of-Use Water Treatment in Rwanda. Bethesda, MD, USA, 2010.
[4] UNICEF Rwanda, WASH Survey in the Districts of Burera, Musanze, Nyabihu & Rubavu Final Results. Kigali, Rwanda, 2011.
[5] National Institute of Statistics of Rwanda, *Population and Housing Census Provisional Results*. Kigali, Rwanda, 2010.
